# Nonclassical roles for IFN-γ and IL-10 in a murine model of immunoedition

**DOI:** 10.2144/fsoa-2019-0108

**Published:** 2020-10-27

**Authors:** Antonela Del Giúdice, Lucas Pagura, María Celeste Capitani, Leandro Ernesto Mainetti, O Graciela Scharovsky, Ricardo José Di Masso, María José Rico, Viviana Rosa Rozados

**Affiliations:** 1 Instituto de Genética Experimental, Facultad de Ciencias Médicas, Universidad Nacional de Rosario, Santa Fe 3100, Rosario 2000, Argentina; 2 CONICET (Consejo Nacional de Investigaciones Científicas y Técnicas) CABA (C1425FQB), Argentina; 3 CIUNR (Consejo de Investigaciones, Universidad Nacional de Rosario) Rosario (2000), Argentina

**Keywords:** cancer immunoediting, mammary adenocarcinoma, mathematical model

## Abstract

**Aim:**

To characterize, by means of univariate and multivariate approaches, the T helper (Th)-1 and Th-2 responses during the different phases of tumor immunoediting.

**Materials & methods:**

We used a multivariate principal component analysis applied to analyze the joint behavior of serum concentrations of IFN-γ, IL-2, IL-10 and IL-4, during the different phases of tumor immunoediting, in CBi/L mice challenged with M-406 mammary adenocarcinoma.

**Results & conclusion:**

Animals in equilibrium phase showed the widest variations in values of the four cytokines. In this experimental model, the role of IFN-γ would be related to tumor growth and progression, while IL-10 would participate in the antitumor immune response.

## Lay abstract

Breast cancer is a complex, multifactor disease that affects about 10% of women in industrialized countries. The immune system has the ability to monitor the appearance of tumors, but the tumors have the ability to escape such rejection. For this reason, in order to design different therapeutic strategies, it is important to know the different mechanisms that take place when a tumor grows or when it is rejected. Here we sought to elucidate some of these mechanisms.

## Background

One of the emerging hallmarks of cancer is immune modulation [1]. The immune system is capable of recognizing and eliminating the transformed cells and, also, is capable of inducing tumor growth. The dual role of the immune system, which is involved in both immunosurveillance and tumor progression, led to the enunciation of the three ‘E’s’ tumor immunoediting theory: elimination (EL), equilibrium (EQ) and escape (ES) [2]. The interaction between tumor cells and the immune system is very complex and involves multiple signals that may be influenced by the expression of cytokines by tumor cells [3], immune cells [4] and other noncancerous cell types such as epithelial cells or cancer-associated fibroblasts [5] of the surrounding tissue. The generation of either a T helper (Th)-1 or (Th)-2 type response depends on the balance among cytokines [6]. There is growing evidence supporting the notion that cytokine expression and T-cell activation play a key role in impairment and deregulation of the immune antitumor and inflammation responses [7]. IL-2 shows a remarkable antitumor potential [8]. On the other hand, several *in vitro* and *in vivo* studies showed, for several types of cancer such as colon, thyroid, breast and lung, that tumor growth could be stimulated by IL-4 through an autocrine loop, inhibition of apoptosis and resistance to death receptors [9,10]. The importance of IFN-γ in an effective antitumor response has been extensively demonstrated [11]. However, in recent years, it was shown that IFN-γ could induce the expression of nonclassical MHC molecules in tumor cells, allowing them to evade lysis mediated by CD8^+^ cytotoxic T lymphocytes [12]. In addition, IFN-γ can induce apoptosis of CD4^+^ cells, essential for the antitumor response carried out by CD8^+^ cytotoxic lymphocytes [13]. IL-10 is commonly associated with the regulation of the antitumor immune response and it was found to play an important role in immune tolerance through the inhibition of cytokine synthesis by activated T cells, natural killer cells and macrophages, in addition to the decrease of macrophages activity as antigen-presenting cells. Tumor cells in melanomas, lung carcinomas and certain types of lymphomas are able to produce IL-10 [14] suggesting that the production of immunosuppressive cytokines such as IL-10 is one of the mechanisms for tumor evasion of immune rejection.

The use of experimental animal models is essential to gain basic understanding of the biological mechanisms underlying human diseases. Tumor cells of rats and mice usually have mutations in genes similar to those that occur in human tumors, suggesting that the mechanisms of control of tumor development might have equivalence among species [15–17]. In the Instituto de Genética Experimental, the CBi/L mouse line was obtained through a divergent selection by body conformation, an experiment developed with the CBi inbred mouse strain [18]. When CBi/L mice are challenged with M-406 mammary adenocarcinoma, the three phases of tumor immunoediting are observed. The tumor begins to grow exponentially in 100% of the animals. After a period of growth, in a group of mice the tumor is rejected until it is completely eliminated; in a second group of mice, the tumor grows exponentially and, in a third group, the tumor enters into a state of equilibrium in which net growth is not appreciated. Then, some tumors of the latter group (EQ) resume exponential growth, escaping from equilibrium and becoming lethal, while the rest of them are rejected until complete elimination [19]. Our objective was to characterize, by means of univariate and multivariate approaches, the Th-1 and Th-2 responses during the different phases of tumor immunoediting using the M-406/CBi/L model in order to understand their role in each immunoediting phase.

## Materials & methods

### Animals

Forty-week-old female CBi/L mice from the Instituto de Genética Experimental, Facultad de Ciencias Médicas, Universidad Nacional de Rosario breeding facilities, were used [19]. Mice were maintained at 23 ± 1°C, on a 12-h-on/12-h-off light cycle and received standard diet (Cargill Laboratory Chow, pelletized) and water *ad libitum*. The animals were treated in accordance to the institutional regulations that complies with the guidelines issued by the Canadian Council on Animal Care [20].

### Tumor

M-406 is a type B semi-differentiated mammary adenocarcinoma, according to Squartini’s classification [21], triple negative (estrogen, progesterone and EGFR-2, negative), that arose spontaneously in an inbred CBi female mouse. It is maintained *in vivo* by serial intraperitoneal passages in syngeneic mice.

### Experimental model

A total of 40 CBi/L female mice were subcutaneous (sc.) challenged with M-406 by trocar (≈8 × 10^5^ cells; day 0). The behavior of tumor bearing animals was daily monitored and when the tumor reached the maximum volume allowed by ethical standards, mice were euthanized by CO_2_ overexposure.

The animals were assigned to one out of three tumor conditions: EL: tumors in regression, between 25–42 days, EQ: tumors which size remained constant for, at least, ten consecutive days, between 12–37 days and ES: tumors with exponential growth, between 37 and 71 days. At the end of the experiment, the animals were anesthetized, blood was drawn by cardiac puncture (cytokine quantification) and samples of tumor were obtained (quantification of lymphocytes in tumor microenvironment).

### Evaluation of tumor volume

Minor and major tumor diameters were measured with a caliper three-times a week, from day 3 on and tumor volume was estimated according to the formula: Vt = (minor diameter)^2^ × major diameter × 0.4.

The data of tumor volume versus time elapsed since tumor challenge, were adjusted to an exponential model: Vt = Start.e∧(k.t), where ‘Start’ is the value of tumor volume (mm^3^) at t = 0, Vt is the tumor volume at t time, t indicates days post-tumor inoculation and k is the exponential growth rate.

### Evaluation of spontaneous metastasis

In order to evaluate the incidence of metastasis and its relationship with the serum concentration of the cytokines studied, we developed an additional experiment in which CBi/L female mice were sc. challenged with M-406, by trocar. The behavior of tumor bearing animals was monitored daily in those animals in which the tumor was in ES (n = 28). When the animals reached the maximum volume ethically permitted, they were anesthetized, blood was drawn by cardiac puncture, their lungs excised and fixed in Bouin solution and the number of metastatic foci determined. The lungs are the main metastatic site of M-406 tumor when growing sc. in CBi/L mice.

### Mononuclear cells conditioned medium

Blood was drawn from anesthetized animals by cardiac puncture with ethylenediaminetetraacetic acid (EDTA) from M-406 bearing CBi/L mice in different phases of tumor growth. Mononuclear cells (Mo) were obtained by Ficoll-Paque PLUS (GE Healthcare, IL, USA) gradient centrifugation. Then 4 × 10^5^ Mo/ml were cultured in RPMI + 10% bovine fetal serum for 24 h at 37° C and 5% CO_2_. The suspension was centrifuged at 2000 r.p.m. for 10 min and the supernatant (MoCM) was separated and stored at -20°C.

### Viability assay

M-406 cells (5 × 10^4^/100 μl) were plated on 96-well culture plates with different MoCM or RPMI (control group). Cells were incubated for 24 h, at 37° C and 5% of CO_2_. Cell viability was evaluated with WST-1 (Cell Proliferation Reagent) kit (Roche, Basilea, Switzerland). The control group value was considered as 100% viability.

### Cytokine quantification

Th-1 (IFN-γ and IL-2) and Th-2 (IL-10 and IL-4) cytokine concentrations were measured by ELISA (BD Biosciences OptEIATM, NJ, USA) following manufacturer instructions, in serum of M-406 bearing mice in the three tumor growth conditions and in MoCM. Quantification was done in triplicate.

### Quantification of lymphocytes in tumor microenvironment

CD4^+^, CD8^+^ and Treg cells were measured by immunohistochemistry in tumor samples of M-406 bearing mice in the three tumor growth conditions. The histological sections of mammary tumors were deparaffinized, incubated overnight at 4°C with anti-CD4 (1:400, Santa Cruz Biotechnology, CA, USA), anti-CD8 (1:400, Santa Cruz Biotechnology) or anti-Foxp3 (1:50, eBioscience, MA, USA) and then, with the Vectastain Elite ABC kit (Vector Labs, USA). Sections were visualized with 3,3′-diaminobenzidine (Sigma, MO, USA) as chromogen. The number of positive cells was calculated in 30 fields at 1000×.

### Expression of cytokines receptors

The expression of IFN-γR (IFN-γ receptor), IL-2R, IL-10R and IL-4R were analyzed in M-406 cells during EQ and ES phases by cellular ELISA assay. M-406 cells were fixed at 10 × 10^5^ cells/well in 96-well plates with 0.5% formaldehyde-buffer for 18 h at 37°C. Then, recombinant IFN-γ (rIFN-γ), rIL-2, rIL-l0 or rIL-4 were added at a saturating concentration (5000 pg/ml for IFN-γ and IL-10; and 10000 pg/ml for IL-2 and IL-4). The plates were incubated 2 h at room temperature, washed, blocked and then incubated for 1 h with, anti-IFN-γ-, anti-IL-2-, anti-IL-10- or anti-IL-4-biotin/streptavidin-peroxidase conjugate. After the addition of tetramethylbenzidine and H_2_O_2_ and 30 min’ incubation at room temperature in the dark, the reaction was stopped with H_2_SO_4_. The optical density was determined at 450–570 nm. All samples were assayed in quadruplicate.

### Statistical analysis

Data obtained were analyzed using Mann–Whitney U test or Kruskal–Wallis one-way analysis of variance by ranks followed by Dunn’s post-test, Student’s t-test, one-way analysis of variance (ANOVA) followed by Tukey’s multiple comparison test and Spearman correlation, as appropriate with GraphPad Prism version 3.0 (GraphPad Software, CA, USA). The joint behavior of the four cytokines was evaluated with the multivariate technique of principal components analysis. p-values lower than 0.05 were considered statistically significant.

## Results

### Classification of animals in the different stages of tumor growth

**Figure 1.**
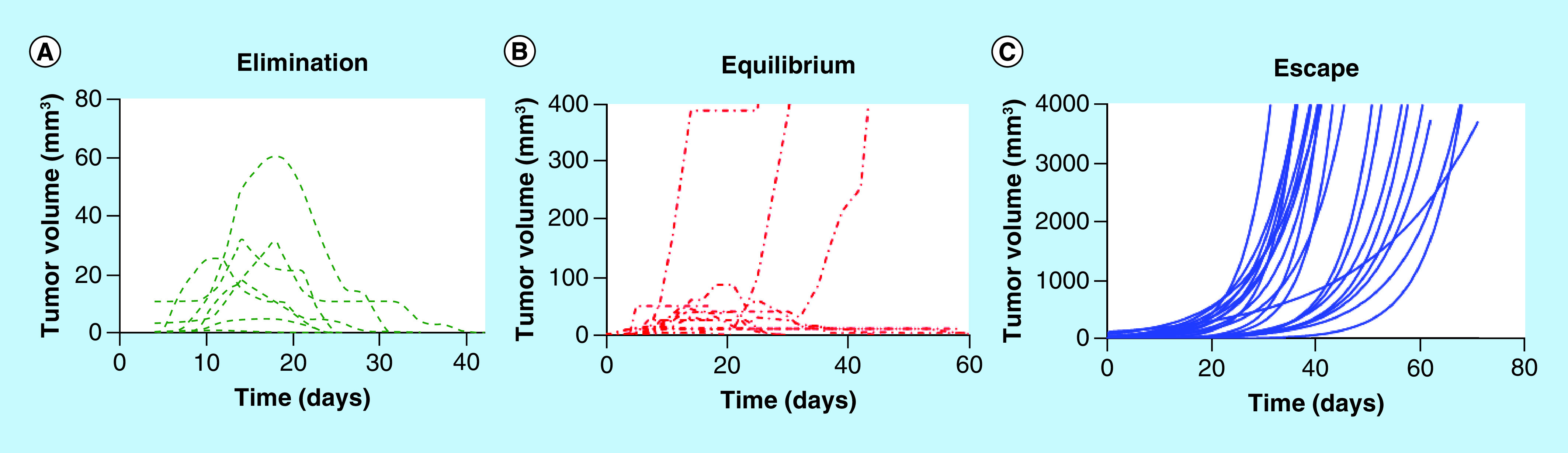
Evolution of tumor growth in CBi/L mice. **(A)** Elimination phase; **(B)** equilibrium phase; **(C)** escape phase.

CBi/L mice challenged with M-406 showed 100% of tumor takes; 22.5% of those tumors were eliminated (EL phase), while 37.5% remained in equilibrium, at least for ten consecutive days (EQ phase) and 40.0% of them grew exponentially (ES phase; Table 1) (Figure 1A, B & C).

**Table 1.** Animals in elimination, equilibrium and elimination phases.

Tumor condition	Animals, % (n/total n)
Take	100% (40/40)
Elimination	22.5% (9/40)
Equilibrium	37.5% (15/40)
Escape	40% (16/40)

### Cytokines serum concentration

**Figure 2.**
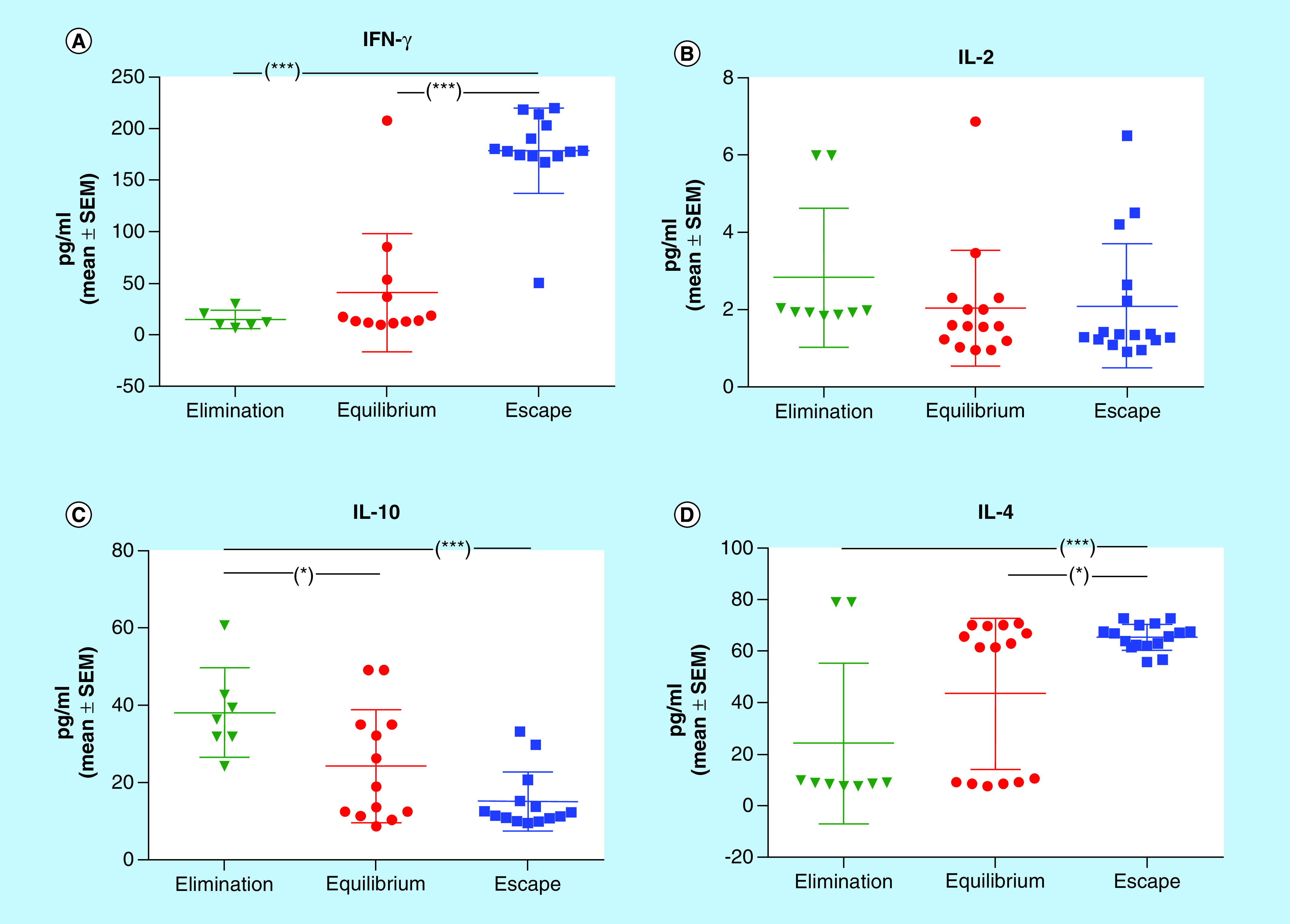
Cytokines serum concentration in different phases of tumor growth. **(A)** IFN-γ; **(B)** IL-2; **(C)** IL-10 and **(D)** IL-4. *p < 0.05; ***p < 0.001 (Kruskal–Wallis and Dunn’s multiple comparisons tests). SEM: Standard error of the mean.

In order to characterize the M-406/CBi/L model with respect to cytokines involved in the different phases of tumor immunoediting, it was evaluated the concentration of Th-1 (IFN-γ, IL-2) and Th-2 (IL-10, IL-4) cytokines in serum from tumor bearing animals during EL, EQ and ES phases (Figure 2).

The IFN-γ concentration was significantly higher in ES (pg/ml; mean ± standard error of the mean (SEM): 178. 60 ± 10. 97) than in EQ (41.10 ± 16.53) and in EL (14.87 ± 3.58; p < 0.001; Figure 2A). IL-2 serum concentration did not show differences among the three phases of tumor growth, ES (2.10 ± 0.40), EQ (2.04 ± 0.39) and EL (2.83 ± 0.58; Figure 2B). The concentration of IL-10 was higher in EL (38.18 ± 4.38) than in EQ (24.23 ± 4.05; p < 0.05) and ES (15.12 ± 2.01; p < 0.001; Figure 2C). The highest level of IL-4 was found in ES (65.37 ± 1.27), followed by EQ (43.48 ± 7.59; p < 0.05) and EL (24.14 ± 10.37; p < 0.001; Figure 2D).

#### Variances comparison

The interleukin concentration data showed highly heterogeneous variances. The behavior of each cytokine was statistically analyzed with the Bartlett’s test of homogeneity of variances in the three phases of tumor growth. While the variations among phases did not reach statistical significance for IL-2 and IL-10, they were highly significant for IFN-γ and IL-4, which showed the highest values in EQ phase (Table 2).

**Table 2.** Variance of cytokines in different phases of tumor growth.

Cytokines	Concentration of cytokines (pg/ml)			Probability
	Elimination	Equilibrium	Escape	
IFN-γ	76.9	3278.7	1686	p = 0.002
IL-2	2.4	2.5	1.3	p = 0.532
IL-10	134,3	213.2	56.8	p = 0.085
IL-4	710.2	909.6	14.7	p < 0.0001

#### Multivariate analysis

**Figure 3.**
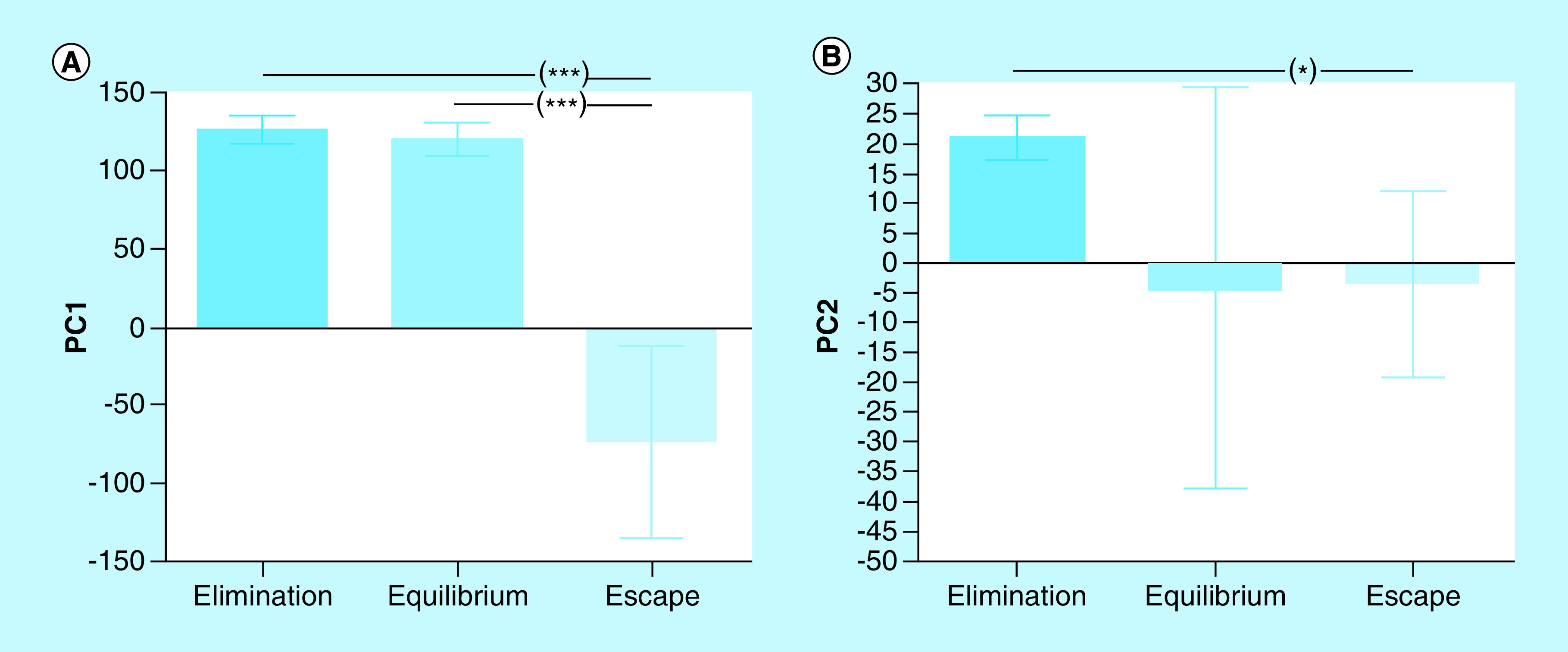
Principal components analysis. *p < 0.05; ***p < 0.001 (ANOVA and Tukey’s multiple comparisons test). ANOVA: Analysis of variance.

**Figure 4.**
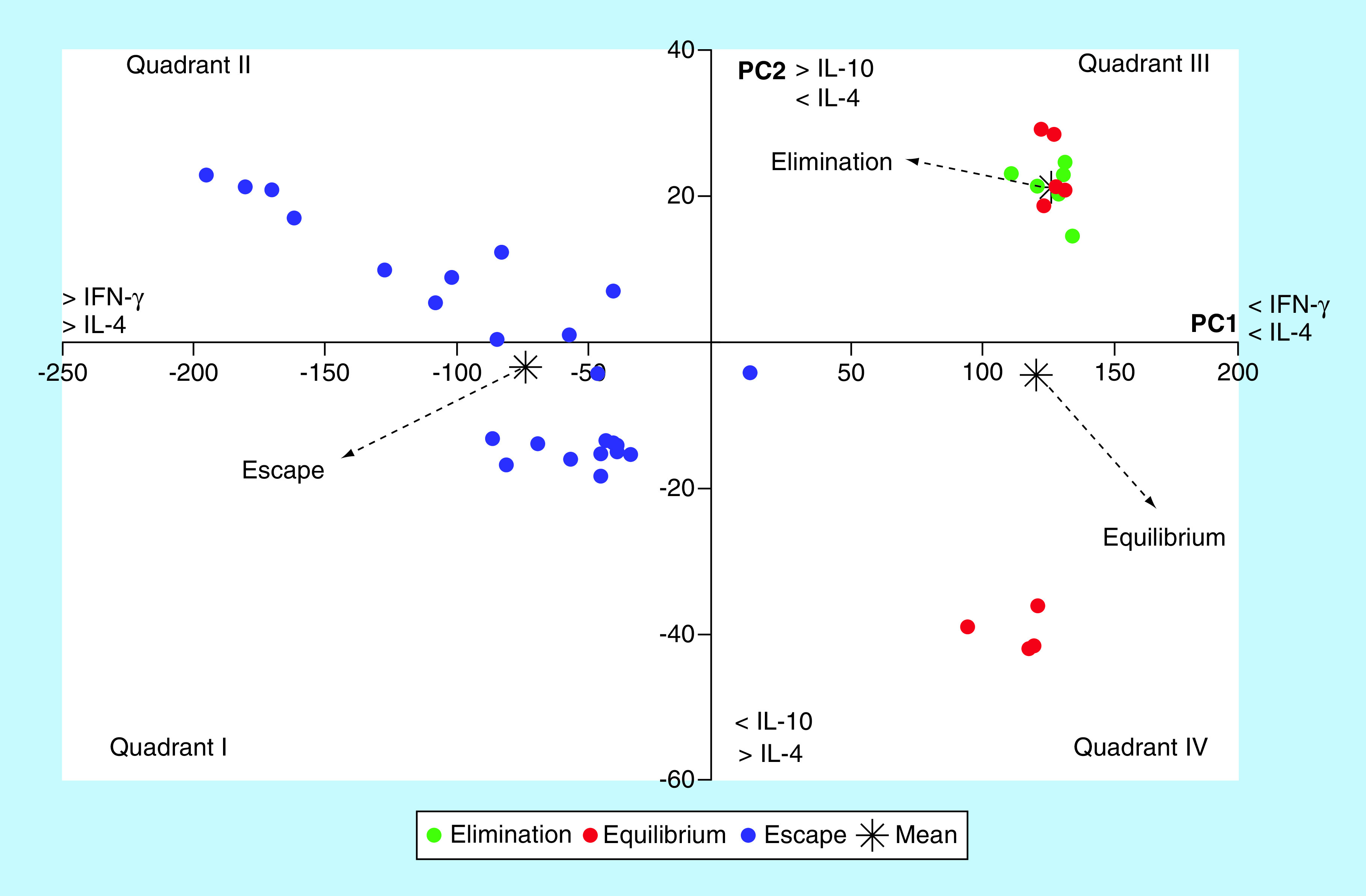
Scatterplot showing the location of three phases of tumor growth in a Cartesian coordinate system of first two principal components.

In order to evaluate the combined action of the four cytokines in each phase of tumor growth, we analyzed them with the multivariate technique of principal components analysis. The two first principal components (PC) explained virtually all the variance in the data (99.28%). The first principal component (PC1) explained the 95.46% of the variance, EL: 126.20 ± 3.55; EQ: 120.40 ± 3.56; ES: -73.62 ± 12.21 (mean ± SEM; Figure 3A); the second principal component (PC2) explained the 3.82%, EL: 21.13 ± 1.47; EQ: -4.49 ± 11.21; ES: -3.45 ± 3.12 (mean ± SEM; Figure 3B). PC1 showed a negative and perfect correlation with IFN-γ (r = -1; p < 0.0001) and, to a lesser extent, with IL-4 (r = -0.699; p < 0.0001). It was not correlated with IL-10 (r = -0.035). PC2 showed positive and significant correlation with IL-10 (r = 0.692; p < 0.0001), negative and significant with IL-4 (r = -0.688; p < 0.0001) and no association with IFN-γ (r = 0.028; p = 0.924). Besides, IL-2 contributed equally to both components. The representation of the values of both components in an orthogonal cartesian system allowed to locate the individuals in different phases of tumor growth (Figure 4).

### CD4^+^, CD8^+^ & Treg lymphocytes in tumor microenvironment

**Figure 5.**
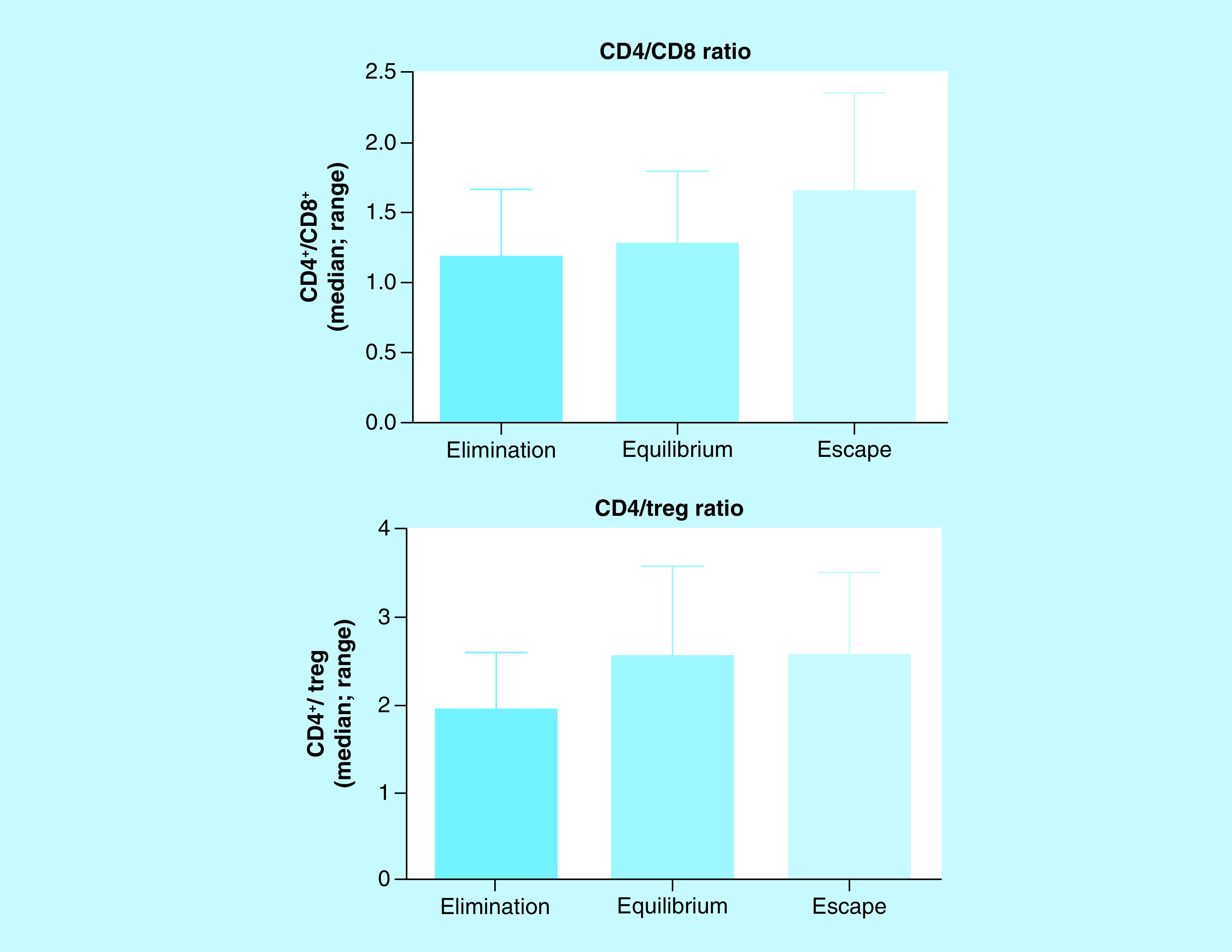
CD4/CD8 and CD4/Treg ratios in tumor microenvironment during different phases of tumor growth. Kruskal–Wallis and Dunn’s multiple comparisons tests.

In the tumor microenvironment, the cells of the immune system interact with each other and with tumor cells. Since the balance between immune cells with different functions determines eventually the effect on target cells, we evaluated the number of CD4^+^, CD8^+^ and Treg cells in tumors going through EL, EQ and ES phases and the CD4/CD8 and CD4/Treg ratios were calculated (Figure 5). When evaluating the ratios, no significant differences were observed between the three phases of tumor immunoediting. CD4^+^/CD8^+^ (number of cells; median, range), EL (1.28 [0.60–1.75]), EQ (1.06 [0.71–2.00]), ES (1.60 [0.50–2.33]); CD4^+^/Treg: EL (2.04 [1.00–2.70]), EQ (2.88 [1.25–3.67]), ES (2.75 [1.00–3.50]).

### Cytokines & metastasis

**Figure 6.**
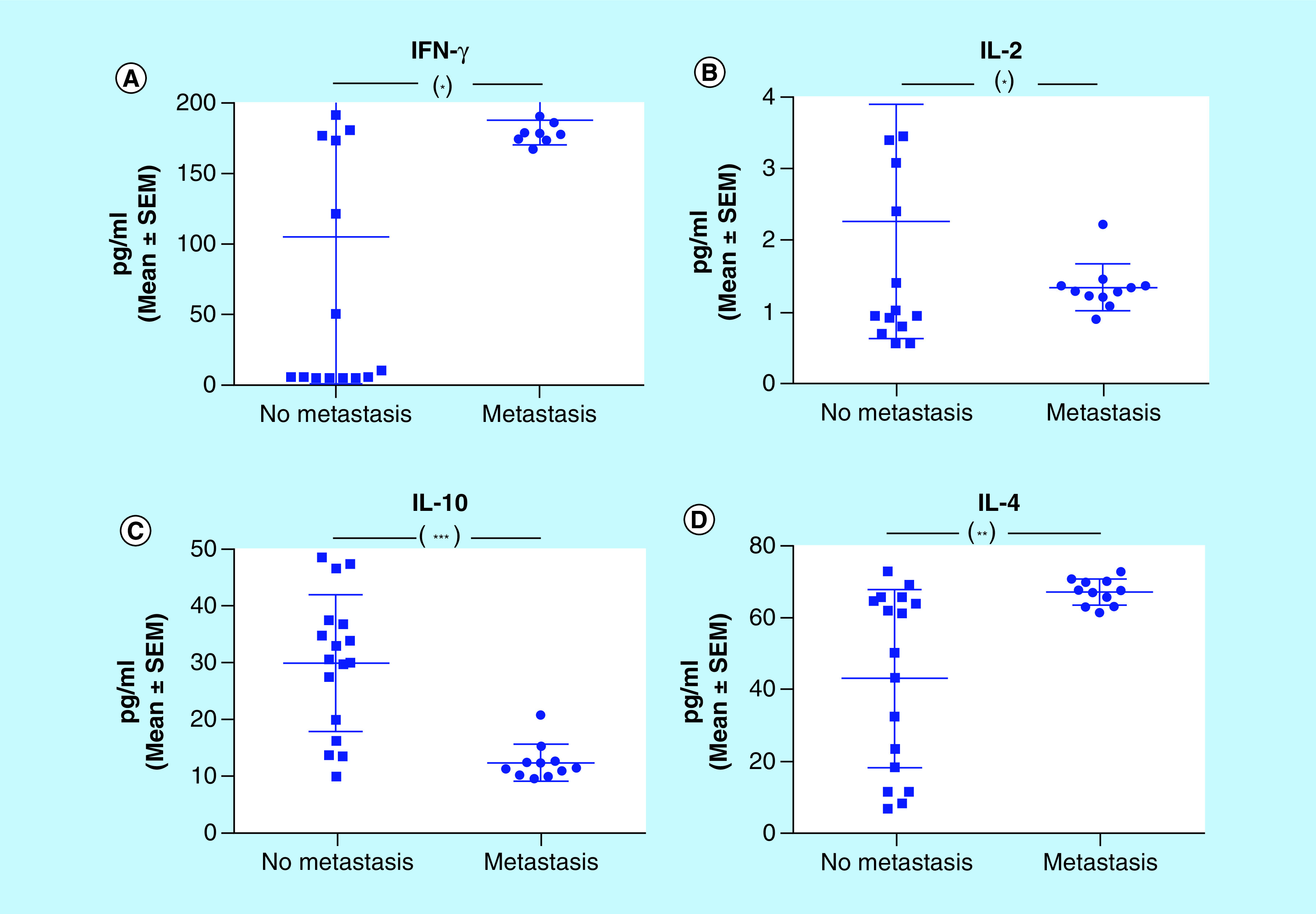
Cytokines serum concentration in animals with and without metastasis (escape phase). **(A)** IFN-γ; **(B)** IL-2; **(C)** IL-10 and **(D)** IL-4. *p < 0.05; **p < 0.01; ***p < 0.001 (unpaired t-test). SEM: Standard error of the mean.

We were interested in evaluating the level of serum cytokines in CBi/L mice during ES phase, according to the development of metastases. Lung metastases were present in 39% (11/28) of the animals. Animals with metastases showed higher levels of IFN-γ (pg/ml, mean ± SEM; 187.50 ± 5.27) and IL-4 (67.11 ± 1.08) than those without metastases (104.60 ± 27.08 and 42.97 ± 6.02, respectively) (Figure 6A & D). On the other hand, animals with metastases evinced lower levels of IL-2 (1.34 ± 0.10) and IL-10 (12.42 ± 0.96) than those with metastases (2.26 ± 0.40 and 29.97 ± 2.91, respectively) (Figure 6B & D).

#### Multivariate analysis

**Figure 7.**
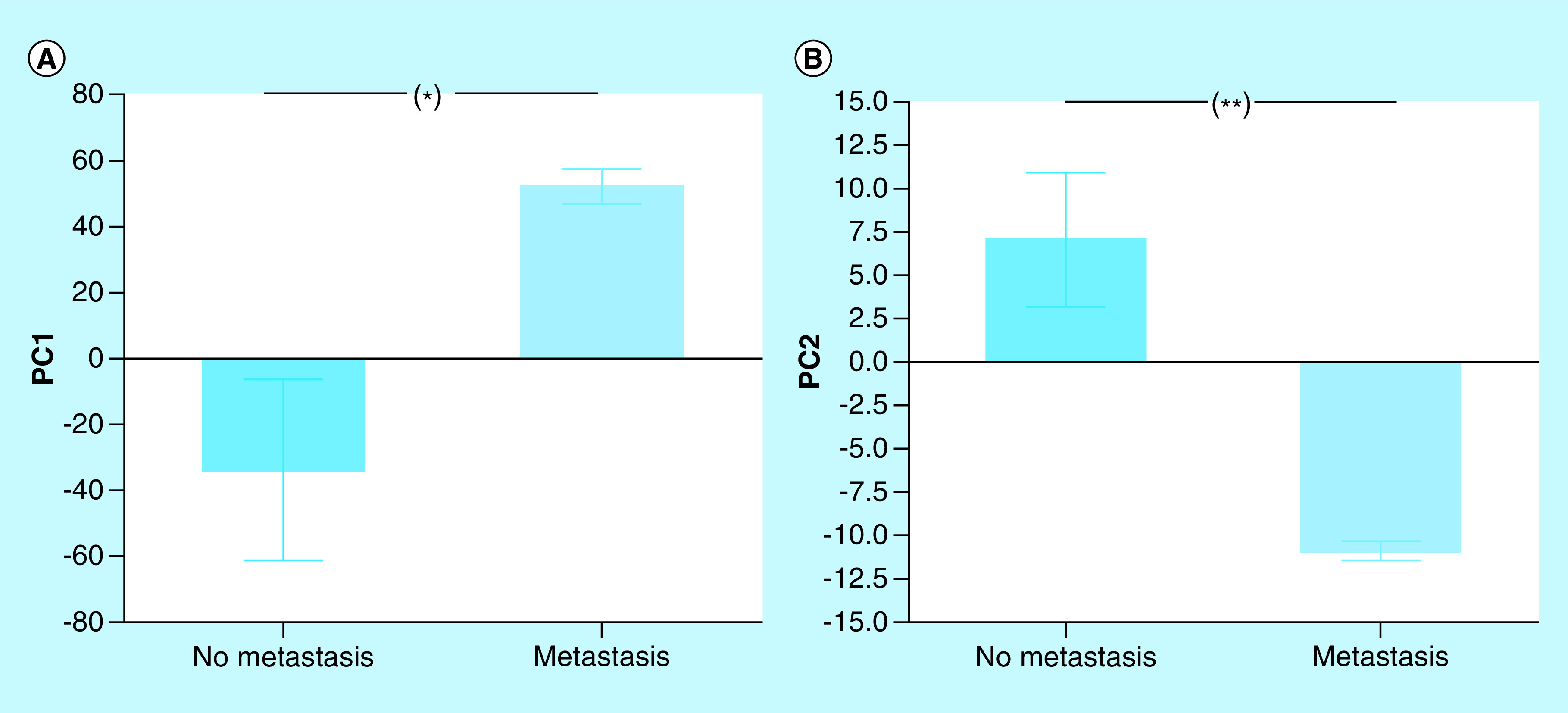
Principal components analysis in animals with and without metastasis. *p < 0.05; **p < 0.01 (unpaired t-test).

**Figure 8.**
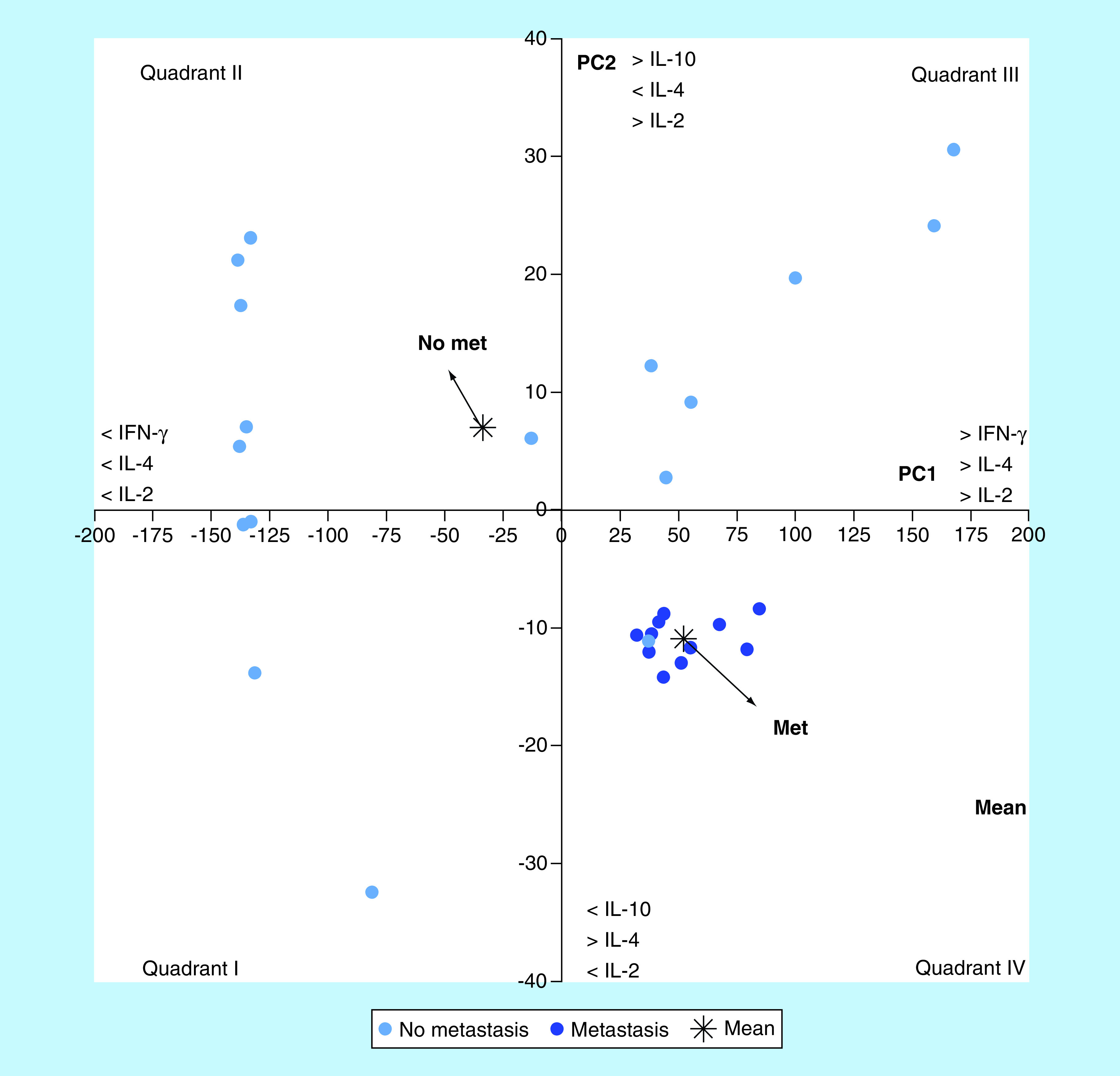
Scatterplot showing the location of the animals with and without metastasis in a Cartesian coordinate system of first two principal components.

The joint analysis of the four ILs with the multivariate technique of principal components, allowed to generate two principal components. The joint analysis of the four ILs with the multivariate technique of PCA, allowed generating two principal components. PC1 explained almost all of the observed variance (96.64%) and showed a positive correlation with the values of IFN-γ (r = 0.999; p < 0.0001) and IL-4 (r = 0.831; p < 0.0001) and, to a lesser extent, with IL-2 (r = 0.478; p = 0.01). This component allowed discriminating (p = 0.01) between animals with metastases (metastases: mean ± SEM, 52.20 ± 5.31) and those without metastases (no metastases: -33.80 ± 27.50). On the other hand, PC2 explained only 2.34% of the variance and correlated positively with IL10 (r = 0.838; p < 0.0001) and not with IFN-γ (r = 0,022; p = 0,914). Animals with (-10.88 ± 0.54) and without (7.04 ± 3.87) metastases also differed (p = 0.0005) in the average values of this component (Figure 7). The representation of PC1 and PC2 values in an orthogonal Cartesian system allowed to locate individuals with metastases in quadrant IV corresponding to negative PC2 and positive PC1 values and those without metastases in quadrant II (negative PC1 and positive PC2 values), highlighting an antagonistic behavior of both groups, regarding the profile of these four cytokines (Figure 8).

### Correlation of MoCM cytokines & M-406 cells viability

**Figure 9.**
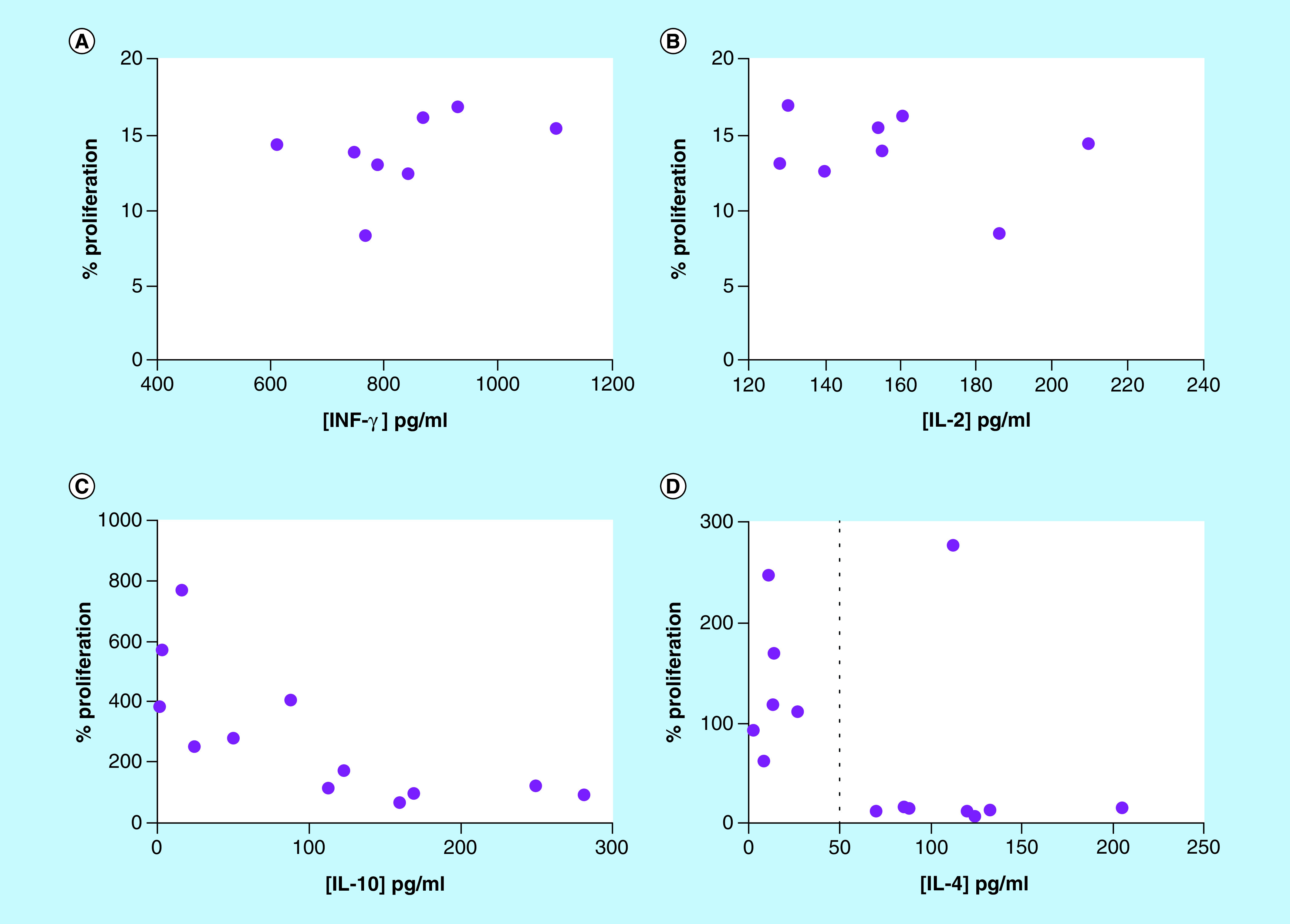
Correlation between M-406 cell proliferation and concentration of different cytokines in MoCM. **(A)** IFN-γ, **(B)** IL-2, **(C)** IL-10: p = 0.0071, r = -0.7292 and **(D)** IL-4 (Pearson correlation).

We evaluated the viability of M-406 cells when incubated with MoCM and determined their correlation with the concentration of each cytokine in the CM. The levels of IFN-γ (Figure 9A), IL-2 (Figure 9B) and IL-4 (Figure 9D) did not correlate with tumor cells viability. On the other hand, there was a negative correlation for IL-10 (r = -0.8252; p = 0.0016; Figure 9C).

### Expression of IFN-γ, IL-2, IL-10 & IL-4 receptors in M-406 cells

**Figure 10.**
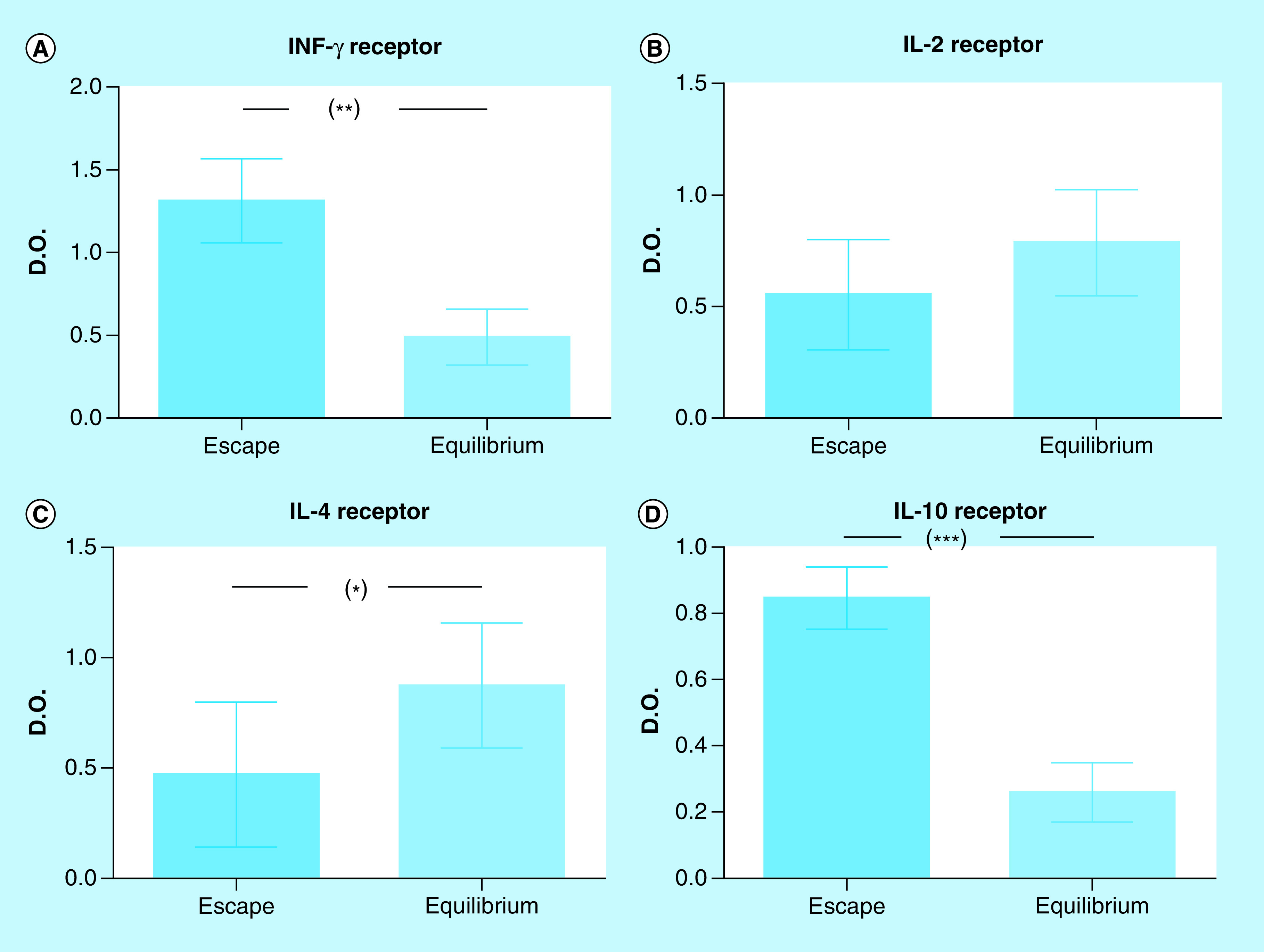
Cytokine receptor concentration in M-406 cells determined by cellular ELISA and expressed as absorbance units (mean ± SEM). *p < 0.05; **p < 0.01; ***p < 0.001 (Student’s t-test).

The expression of IFN-γR and IL-10R in M-406 cells, evaluated with the cellular ELISA assay, was higher in ES than in EQ phases (p < 0.001 and p < 0.01, respectively) (Figure 10A & C), while IL-4R showed higher values in EQ than in ES phase (p < 0.05; Figure 10D). No significant differences were observed for IL-2R (Figure 10B).

## Discussion

For a long time, there was uncertainty about the role of the immune system on tumor development. Work from many labs has unequivocally documented in animal tumor models and also in humans, that immunity can, in fact, not only prevent or control tumor growth but also facilitate it. This dynamic process, whereby the immune system not only protects against cancer development but also shapes the immunogenicity of emerging tumors is composed of three phases: elimination, equilibrium and escape [22]. The study of each of these phases has clarified the understanding of the complex relationship between the immune system and tumorigenesis. The ability of cancer cells to evade the antitumor immune response is now recognized as one of the hallmarks of cancer.

The mouse is an ideal model organism for studying human diseases, due to their genetic similarity and the wide knowledge of mouse biology. For this reason, there has been a mounting interest toward the generation of experimental models able to reproduce the main features associated with different diseases. The CBi/L inbred mouse line, generated at the Instituto de Genética Experimental, jointly with the M-406 mammary adenocarcinoma, provides a suitable model for studying the three ‘E’s’ of tumor immunoediting [19].

Different types of cells integrate solid tumors, among which, apart from the heterogeneous tumor cells themselves, can be found cells from diverse origins such as stem, stromal, endothelial and a wide range of immune cells [23]. The balance within subpopulations of immune cells is an important factor in tumor progression and the presence of different cytokines coordinate the action of these cells. This knowledge led us to evaluate the concentration of Th-1 (IFN-γ, IL-2) and Th-2 (IL-10, IL-4) serum cytokines in M-406 bearing CBi/L mice, in the different stages of tumor immunoediting.

In accordance to previous observations, when CBi/L mice were challenged with M-406, we observed 22.5, 37.5 and 40.0% of animals in EL, EQ and ES phases, respectively, supporting the M-406/CBi/L system as a good murine model to study the process of tumor immunoediting [19].

Breast cancer is affected, like other tumors, by the immune system and the cytokines produced by immune cells play different roles in its initiation, progression or regression. The analysis of different cytokines in the three phases of tumor growth did not show, in our model, a classic Th-1 or Th-2 response.

The dynamics and roles of CD4^+^, CD8^+^ and Tregs cells in the pathogenesis of breast cancer remain unclear. It is established that an effective antitumor immune response requires the involvement of both CD4^+^ and CD8^+^ T cells [24]. On the other side, it has been shown that tumor-infiltrating Treg cells-induced immunosuppressive microenvironment prevents effective antitumor immunity and becomes a major obstacle to the success of immunotherapy against breast cancer [25]. The tumor-infiltrating cells have distinct roles for control of tumor progression and clinical outcomes. Huang *et al.*, observed in murine models, that the proportion of CD8^+^ T cells was more dominant than CD4^+^ T cells in tumor-infiltrating lymphocytes (TILs) in the early stages of tumor development. However, CD4^+^ T cells infiltrated more rapidly the tumor sites than CD8^+^ T cells during the tumor progression and, therefore, they became the dominant population in late stages of tumor development [26]. The preliminary study, carried out in CBi/L and M-406 tumor model, did not show differences in CD4^+^/CD8^+^ or CD4^+^/Treg ratios, in the tumor samples obtained at the end of the experiment (Figure 5), suggesting the need to study the evolution of such values during tumor growth, more than having the picture in a fixed time point. These results prompt us to study, in the near future, the kinetics of immune cells participation in tumor microenvironment during the immunoediting phases.

The concentration of IFN-γ found in ES phase did not agree with the antitumor function classically assigned to this cytokine (Figure 2A). The levels of serum IFN-γ and tumor expression of IFN-γR were higher in animals in ES phase than those in EQ (Figure 10A). Our results are in accordance with those obtained in clinical trials, in which the administration of IFN-γ did not elicit an antitumor response [27] or, even more, produced an unfavorable response [28].

IL-2, a fundamental factor for growth and differentiation of T lymphocytes, plays a dominant role in the regulation of the immune response [29,30]. However, in our tumor model, the serum concentrations of IL-2 (Figure 2B) and the expression of IL-2R (Figure 10B) did not show differences among the three phases. Moreover, the different MoCM IL-2 concentrations did not correlate with tumor cells viability (Figure 9B).

The results obtained for IL-10 showed the higher concentration at EL phase, compared with ES and EQ phases (Figure 2C). In addition, according to the mentioned results, the viability of M-406 cells was low, when incubated with MoCM containing high concentrations of IL-10 (Figure 9C), suggesting an antitumor role for this cytokine. MacNeil *et al.* and Chen *et al.* observed that IL-10 could stimulate the differentiation and growth of CD8^+^ lymphocytes, indicating an immunostimulatory rol [31,32]. On the contrary, Zou *et al.* found, in gastric cancer patients, elevated plasma levels of IL-10 associated with increased tumor size [33].

IL-4 is a typical pleiotropic Th2 cytokine, which was found to directly induce tumor cell proliferation and to mediate resistance to apoptosis, supporting tumor growth [34,35]. Our own results agree with those observations, since we detected higher IL-4 serum concentrations in animals in ES phase (Figure 2D). However, the *in vitro* assays showed that M-406 cells viability was low, when incubated with MoCM containing concentrations of IL-4 higher than 50 pg/ml (Figure 9C). On the other hand, the expression of IL-4R on M-406 cells in ES phase was lower than that in EQ phase (Figure 10C). These apparently contradictory results could have an explanation through the production of the soluble form of the IL-4R (sIL-4R) [36]. If that would be the case, it would explain the fact that, even with high concentration of IL-4, the viability of tumor cells decreases, because the sIL-4R would bind IL-4, hence diminishing its concentration in the tumor microenvironment. It also would explain the low concentration of IL-4R in the tumor cells caused either by the increased proteolysis of the membrane-bound receptor [37] or by the expression of the splice variant [38]. Future studies will shed some light on this matter.

Principal component analysis is a multivariate statistical technique of synthesis of information or reduction of the number of variables. It has the potential to expose nonobvious relationships between characters, thus contributing to a more efficient interpretation of the information contained in a set of data. Its application to the analysis of the joint behavior of IFN-γ, IL-2, IL-10 and IL-4 showed that the differences observed in the growth of M-406 tumor could be explained by the first two main components. The first component showed a perfect and negative association with IFN-γ and, to a lesser extent, with IL-4. On the contrary, the second component showed a positive association with IL-10 and a negative association with IL-4 (Figures 3 & 4). The graphic representation of the individual values of both components in an orthogonal cartesian system allowed to observe differences in the quadrant of location and also in their dispersion. Individuals with tumors in EL phase were located in quadrant III (>IL-10, <IFN-γ and <IL-4), while those with tumors in ES phase and those in EQ phase, were found, in average, in quadrants I (<IL-10, >IFN-γ and >IL-4) and IV (<IL-10, <IFN-γ and <IL-4), respectively. While values of individuals bearing tumors in EL phase showed a very low dispersion around the mean value, those with tumor in ES phase were dispersed in quadrants I, II and IV and those animals with tumor in EQ phase were distributed in quadrants III and IV. This difference in the variance pattern could be interpreted as evidence that animals with tumors in different phases are developing differential antitumor immune responses although they are bearing the same type of tumor.

The malignancy of a tumor is fundamentally associated with the development of metastatic lesions. For the development of metastases, such as for tumorigenesis and tumor progression, several hypotheses have emerged [39]. The inflammation in the tumor microenvironment is due to the presence of immune cells and specific inflammatory mediators, including growth factors, cytokines and chemokines, which act as immunoregulators in the development of metastasis [39–41].

The multivariate analysis of IFN-γ, IL-2, IL-10 and IL-4 in animals with M-406 in ES phase with or without metastasis, showed that the two first principal component explained a great proportion of the overall observed variance (Figure 7). The representation of the values of both components in an orthogonal Cartesian system allowed to observe a very low dispersion of values in individuals with metastasis which were strongly clustered in quadrant IV (<IL-10; >IL-4; <IL-2), while those without metastases were located on average in quadrant II (<IFN-γ; <IL-4; <IL-2; Figure 8). On the contrary, in a model of experimental metastasis of renal adenocarcinoma, the treatment with a combination of IFN-γ and IL-2 had the most powerful inhibitory effect on pulmonary metastasis. Our own results differ from these ones. Nevertheless, it must be taken into consideration that both experimental models differ in several aspects [42]. Similarly, in highly metastatic H7 murine pancreatic adenocarcinoma cells, it was observed that tumor cells stimulated IFN-γ secretion by host cells which, in turn, stimulated nitric oxide production and suppressed tumor growth and metastasis [43].

IL-10 exerts a complex role in breast cancer initiation and progression [44,45]. In our models, we found that the animals with metastasis showed lower value of IL-10 compared with the animals without metastasis. Similarly, Autenshlyus *et al.* found that the concentration of IL-10 was lower in supernatant of cultured mammary adenocarcinoma cells from patients with regional lymph node metastasis as compared with patients without them. These data indicate that IL-10 can play the suppressor role in the cytokine-dependent mechanism underlying metastasis to regional lymph nodes [46].

## Conclusion

The analysis of the dispersion shown by the concentrations of the four cytokines studied led to the interesting finding that the widest variations corresponded to animals traversing the EQ phase (Table 2). This result suggests coexistence in this stage of individuals with levels of ILs compatible with both ES and EL phases. Thus, a CBi/L mouse with a tumor in EQ phase and high levels of IFN-γ and IL-4 will be prone to resume growth and eventually enter into the ES phase. In addition, in this phase, the animals with metastasis showed lower concentration of IL-10 and higher of IFN-γ compared with the animals without metastasis. On the contrary, high levels of IL-10 and low levels of IFN-γ and IL-4 will lead to EL phase. Therefore, in our experimental model, IFN-γ would favor tumor growth and progression and, on the other hand, IL-10 would participate in the antitumor immune response. These interesting results underline the fact that not every tumor model is associated with a typical Th-1/Th-2 response. Moreover, the diversity of behaviors shown by the different tumor models that have been utilized up to now points to the necessity of characterizing each one without assuming already established patterns that, as in our case, do not follow the expected response. Future studies should investigate the possibility of finding general markers that could enable prediction of the behavior of each tumor model system.

## Future perspective

The study of the different factors involved in the antitumor immune response has allowed design of different therapeutic strategies. Hence, we propose to study other molecules and cells in this tumor model in order to better understand the dual function of the immune system during the cancer initiation and development.

**Summary points**

- The analysis of different cytokines in the three phases of tumor growth (escape [ES], equilibrium and elimination), in CBi/L mice challenged with M-406 mammary adenocarcinoma did not show a classic Th-1 or Th-2 response.
- The preliminary study, carried out in tumor samples obtained in the three phases of tumor growth, did not show differences in CD4^+^/CD8^+^ or CD4^+^/Treg ratios.
- The interleukin concentration data showed highly heterogeneous variances, corresponding to the widest variations to animals undergoing the equilibrium phase. This result suggests the coexistence in this stage of individuals with levels of ILs compatible with both ES and elimination phases.
- The application of the multivariate statistical analysis of the joint behavior of IFN-γ, IL-2, IL-10 and IL-4 showed that the differences observed in M-406 tumor growth could be explained by the first two main components.
- The first component showed a perfect and negative association with IFN-γ and, to a lesser extent, with IL-4. On the contrary, the second component showed a positive association with IL-10 and a negative association with IL-4.
- The multivariate analysis of IFN-γ, IL-2, IL-10 and IL-4 in M-406 bearing animals with or without metastasis going through ES phase, showed that the two first principal component explained a high proportion of the overall observed variance.
- Animals with metastases showed a lower concentration of IL-10 and higher of IFN-γ, compared with those without metastases.
- In CBi/L mouse challenged with M-406 tumor, IFN-γ would favor tumor growth and progression and, on the other hand, IL-10 would participate in the antitumor immune response.
- These interesting results underline the fact that not every tumor model is associated with a typical Th-1/Th-2 response.
